# THE CONCISE GUIDE TO PHARMACOLOGY 2021/22: Introduction and Other Protein Targets

**DOI:** 10.1111/bph.15537

**Published:** 2021-09-16

**Authors:** Stephen P H Alexander, Eamonn Kelly, Alistair Mathie, John A Peters, Emma L Veale, Jane F Armstrong, Elena Faccenda, Simon D Harding, Adam J Pawson, Christopher Southan, O Peter Buneman, John A Cidlowski, Arthur Christopoulos, Anthony P Davenport, Doriano Fabbro, Michael Spedding, Jörg Striessnig, Jamie A Davies, Katelin E. Ahlers‐Dannen, Mohammed Alqinyah, Thiruma V. Arumugam, Christopher Bodle, Josephine Buo Dagner, Bandana Chakravarti, Shreoshi P. Choudhuri, Kirk M. Druey, Rory A. Fisher, Kyle J. Gerber, John R. Hepler, Shelley B. Hooks, Havish S. Kantheti, Behirda Karaj, Somayeh Layeghi‐Ghalehsoukhteh, Jae‐Kyung Lee, Zili Luo, Kirill Martemyanov, Luke D. Mascarenhas, Harrison McNabb, Carolina Montañez‐Miranda, Osita Ogujiofor, Hoa Phan, David L. Roman, Vincent Shaw, Benita Sjogren, Christopher Sobey, Mackenzie M. Spicer, Katherine E. Squires, Laurie Sutton, Menbere Wendimu, Thomas Wilkie, Keqiang Xie, Qian Zhang, Yalda Zolghadri

**Affiliations:** ^1^ School of Life Sciences University of Nottingham Medical School Nottingham NG7 2UH UK; ^2^ School of Physiology, Pharmacology and Neuroscience University of Bristol Bristol BS8 1TD UK; ^3^ School of Engineering, Arts, Science and Technology University of Suffolk Ipswich IP4 1QJ UK; ^4^ Neuroscience Division Medical Education Institute, Ninewells Hospital and Medical SchoolUniversity of Dundee Dundee DD1 9SY UK; ^5^ Medway School of Pharmacy The Universities of Greenwich and Kent at Medway, Anson Building, Central Avenue, Chatham Maritime Chatham, Kent ME4 4TB UK; ^6^ Centre for Discovery Brain Sciences University of Edinburgh Edinburgh EH8 9XD UK; ^7^ Laboratory for Foundations of Computer Science School of InformaticsUniversity of Edinburgh Edinburgh EH8 9LE UK; ^8^ National Institute of Environmental Health Sciences, National Institutes of Health, Department of Health and Human Services Research Triangle Park NC 27709 USA; ^9^ Monash Institute of Pharmaceutical oxPharmaceutical Sciences and Department of Pharmacology Monash University Parkville Victoria 3052 Australia; ^10^ Clinical Pharmacology Unit University of Cambridge Cambridge CB2 0QQ UK; ^11^ PIQUR Therapeutics Basel 4057 Switzerland; ^12^ Spedding Research Solutions SARL Le Vésinet 78110 France; ^13^ Pharmacology and Toxicology, Institute of Pharmacy University of Innsbruck A‐6020 Innsbruck Austria; ^14^ University of Iowa Iowa City USA; ^15^ University of Georgia Athens USA; ^16^ National University of Singapore Singapore Singapore; ^17^ University of Pittsburgh Iowa City USA; ^18^ University of Texas Southwestern Medical Center Dallas USA; ^19^ National Institute of Health Bethesda USA; ^20^ Tetracore, Inc. Athens USA; ^21^ Emory University Athens USA; ^22^ University of Texas Southwestern Dallas USA; ^23^ University of Michigan East Lansing USA; ^24^ Cobel Darou Shiraz Iran; ^25^ Scripps Research Institute Jupiter USA; ^26^ Purdue University West Lafayette USA; ^27^ Michigan State University East Lansing USA; ^28^ La Trobe University Clayton Australia; ^29^ University of Maryland Jupiter USA

## Abstract

The Concise Guide to PHARMACOLOGY 2021/22 is the fifth in this series of biennial publications. The Concise Guide provides concise overviews, mostly in tabular format, of the key properties of nearly 1900 human drug targets with an emphasis on selective pharmacology (where available), plus links to the open access knowledgebase source of drug targets and their ligands (www.guidetopharmacology.org), which provides more detailed views of target and ligand properties. Although the Concise Guide constitutes over 500 pages, the material presented is substantially reduced compared to information and links presented on the website. It provides a permanent, citable, point‐in‐time record that will survive database updates. The full contents of this section can be found at http://onlinelibrary.wiley.com/doi/bph.15537. In addition to this overview, in which are identified ‘Other protein targets’ which fall outside of the subsequent categorisation, there are six areas of focus: G protein‐coupled receptors, ion channels, nuclear hormone receptors, catalytic receptors, enzymes and transporters. These are presented with nomenclature guidance and summary information on the best available pharmacological tools, alongside key references and suggestions for further reading. The landscape format of the Concise Guide is designed to facilitate comparison of related targets from material contemporary to mid‐2021, and supersedes data presented in the 2019/20, 2017/18, 2015/16 and 2013/14 Concise Guides and previous Guides to Receptors and Channels. It is produced in close conjunction with the Nomenclature and Standards Committee of the International Union of Basic and Clinical Pharmacology (NC‐IUPHAR), therefore, providing official IUPHAR classification and nomenclature for human drug targets, where appropriate.

## Conflict of interest

The authors state that there are no conflicts of interest to disclose.

## Table of contents

**S1 Introduction and Other Protein Targets**

S8 Adiponectin receptors

S9 Aryl hydrocarbon receptor

S10 Non‐enzymatic BRD containing proteins

S11 CD molecules

S13 Methyllysine reader proteins

S14 Fatty acid‐binding proteins

S16 Notch receptors

S17 Regulators of G protein Signaling (RGS) proteins

S17 RZ family

S18 R4 family

S19 R7 family

S19 R12 family

S20 Sigma receptors

S21 Transthyretin

S22 Tubulins

S23 SARS‐CoV‐2

S23 Structural proteins

S24 Polyproteins

S24 Proteases

S25 Nucleic acid turnover

S25 Other proteins

**S27 G protein‐coupled receptors**

S31 Orphan and other 7TM receptors

S32 Class A Orphans

S41 Class C Orphans

S41 Opsin receptors

S42 Taste 1 receptors

S43 Taste 2 receptors

S44 Other 7TM proteins

S45 5‐Hydroxytryptamine receptors

S48 Acetylcholine receptors (muscarinic)

S50 Adenosine receptors

S52 Adhesion Class GPCRs

S55 Adrenoceptors

S59 Angiotensin receptors

S60 Apelin receptor

S61 Bile acid receptor

S62 Bombesin receptors

S63 Bradykinin receptors

S64 Calcitonin receptors

S66 Calcium‐sensing receptor

S67 Cannabinoid receptors

S68 Chemerin receptors

S69 Chemokine receptors

S73 Cholecystokinin receptors

S74 Class Frizzled GPCRs

S76 Complement peptide receptors

S78 Corticotropin‐releasing factor receptors

S79 Dopamine receptors

S81 Endothelin receptors

S82 G protein‐coupled estrogen receptor

S83 Formylpeptide receptors

S84 Free fatty acid receptors

S86 GABAB receptors

S87 Galanin receptors

S89 Ghrelin receptor

S90 Glucagon receptor family

S91 Glycoprotein hormone receptors

S92 Gonadotrophin‐releasing hormone receptors

S93 GPR18, GPR55 and GPR119

S94 Histamine receptors

S96 Hydroxycarboxylic acid receptors

S97 Kisspeptin receptor

S98 Leukotriene receptors

S100 Lysophospholipid (LPA) receptors

S101 Lysophospholipid (S1P) receptors

S103 Melanin‐concentrating hormone receptors

S104 Melanocortin receptors

S105 Melatonin receptors

S106 Metabotropic glutamate receptors

S108 Motilin receptor

S110 Neuromedin U receptors

S111 Neuropeptide FF/neuropeptide AF receptors

S112 Neuropeptide S receptor

S113 Neuropeptide W/neuropeptide B receptors

S114 Neuropeptide Y receptors

S116 Neurotensin receptors

S117 Opioid receptors

S119 Orexin receptors

S120 Oxoglutarate receptor

S120 P2Y receptors

S123 Parathyroid hormone receptors

S124 Platelet‐activating factor receptor

S125 Prokineticin receptors

S126 Prolactin‐releasing peptide receptor

S127 Prostanoid receptors

S129 Proteinase‐activated receptors

S131 QRFP receptor

S132 Relaxin family peptide receptors

S134 Somatostatin receptors

S135 Succinate receptor

S136 Tachykinin receptors

S137 Thyrotropin‐releasing hormone receptors

S138 Trace amine receptor

S139 Urotensin receptor

S140 Vasopressin and oxytocin receptors

S142 VIP and PACAP receptors

**S157 Ion channels**

S159 Ligand‐gated ion channels

S160 5‐HT_3_ receptors

S162 Acid‐sensing (proton‐gated) ion channels (ASICs)

S165 Epithelial sodium channel (ENaC)

S166 GABA_*A*_ receptors

S172 Glycine receptors

S175 Ionotropic glutamate receptors

S180 IP_3_ receptors

S181 Nicotinic acetylcholine receptors

S185 P2X receptors

S187 ZAC

S188 Voltage‐gated ion channels

S188 CatSper and Two‐Pore channels

S190 Cyclic nucleotide‐regulated channels

S192 Potassium channels

S193 Calcium‐ and sodium‐activated potassium channels

S195 Inwardly rectifying potassium channels

S199 Two‐pore domain potassium channels

S201 Voltage‐gated potassium channels

S204 Ryanodine receptors

S205 Transient Receptor Potential channels

S219 Voltage‐gated calcium channels

S222 Voltage‐gated proton channel

S223 Voltage‐gated sodium channels

S225 Aquaporins

S227 Chloride channels

S228 ClC family

S230 CFTR

S231 Calcium activated chloride channel

S232 Maxi chloride channel

S233 Volume regulated chloride channels

S234 Connexins and Pannexins

S236 Piezo channels

S237 Sodium leak channel, non‐selective

S238 Orai channels

**S245 Nuclear hormone receptors**

S247 1A. Thyroid hormone receptors

S248 1B. Retinoic acid receptors

S249 1C. Peroxisome proliferator‐activated receptors

S250 1D. Rev‐Erb receptors

S251 1F. Retinoic acid‐related orphans

S252 1H. Liver X receptor‐like receptors

S253 1I. Vitamin D receptor‐like receptors

S254 2A. Hepatocyte nuclear factor‐4 receptors

S255 2B. Retinoid X receptors

S255 2C. Testicular receptors

S256 2E. Tailless‐like receptors

S256 2F. COUP‐TF‐like receptors

S257 3B. Estrogen‐related receptors

S257 4A. Nerve growth factor IB‐like receptors

S258 5A. Fushi tarazu F1‐like receptors

S259 6A. Germ cell nuclear factor receptors

S259 0B. DAX‐like receptors

S260 Steroid hormone receptors

S260 3A. Estrogen receptors

S261 3C. 3‐Ketosteroid receptors

**S264 Catalytic receptors**

S266 Cytokine receptor family

S266 IL‐2 receptor family

S268 IL‐3 receptor family

S268 IL‐6 receptor family

S270 IL‐12 receptor family

S271 Prolactin receptor family

S272 Interferon receptor family

S273 IL‐10 receptor family

S274 Immunoglobulin‐like family of IL‐1 receptors

S275 IL‐17 receptor family

S276 GDNF receptor family

S277 Integrins

S281 Pattern recognition receptors

S281 Toll‐like receptor family

S283 NOD‐like receptor family

S285 RIG‐I‐like receptor family

S285 Receptor guanylyl cyclase (RGC) family

S286 Transmembrane guanylyl cyclases

S287 Nitric oxide (NO)‐sensitive (soluble) guanylyl cyclase

S288 Receptor tyrosine kinases (RTKs)

S289 Type I RTKs: ErbB (epidermal growth factor) receptor family

S290 Type II RTKs: Insulin receptor family

S291 Type III RTKs: PDGFR, CSFR, Kit, FLT3 receptor family

S292 Type IV RTKs: VEGF (vascular endothelial growth factor) receptor family

S293 Type V RTKs: FGF (fibroblast growth factor) receptor family

S294 Type VI RTKs: PTK7/CCK4

S294 Type VII RTKs: Neurotrophin receptor/Trk family

S295 Type VIII RTKs: ROR family

S296 Type IX RTKs: MuSK

S296 Type X RTKs: HGF (hepatocyte growth factor) receptor family

S297 Type XI RTKs: TAM (TYRO3‐, AXL‐ and MER‐TK) receptor family

S297 Type XII RTKs: TIE family of angiopoietin receptors

S298 Type XIII RTKs: Ephrin receptor family

S299 Type XIV RTKs: RET

S299 Type XV RTKs: RYK

S300 Type XVI RTKs: DDR (collagen receptor) family

S300 Type XVII RTKs: ROS receptors

S301 Type XVIII RTKs: LMR family

S301 Type XIX RTKs: Leukocyte tyrosine kinase (LTK) receptor family

S302 Type XX RTKs: STYK1

S302 Receptor serine/threonine kinase (RSTK) family

S303 Type I receptor serine/threonine kinases

S304 Type II receptor serine/threonine kinases

S304 Type III receptor serine/threonine kinases

S305 RSTK functional heteromers

S306 Receptor tyrosine phosphatase (RTP) family

S308 Tumour necrosis factor (TNF) receptor family

**S313 Enzymes**

S318 Acetylcholine turnover

S318 Adenosine turnover

S321 Amino acid hydroxylases

S322 L‐Arginine turnover

S336 2.1.1.‐ Protein arginine N‐methyltransferases

S322 Arginase

S323 Arginine:glycine amidinotransferase

S323 Dimethylarginine dimethylaminohydrolases

S324 Nitric oxide synthases

S325 Carbonic anhydrases

S325 Carboxylases and decarboxylases

S326 Carboxylases

S327 Decarboxylases

S328 Catecholamine turnover

S330 Ceramide turnover

S331 Serine palmitoyltransferase

S331 Ceramide synthase

S332 Sphingolipid Δ4‐desaturase

S332 Sphingomyelin synthase

S333 Sphingomyelin phosphodiesterase

S333 Neutral sphingomyelinase coupling factors

S334 Ceramide glucosyltransferase

S334 Acid ceramidase

S334 Neutral ceramidases

S335 Alkaline ceramidases

S335 Ceramide kinase

S336 Chromatin modifying enzymes

S336 2.1.1.‐ Protein arginine N‐methyltransferases

S337 3.5.1.‐ Histone deacetylases (HDACs)

S338 Cyclic nucleotide turnover/signalling

S338 Adenylyl cyclases (ACs)

S340 Exchange protein activated by cyclic AMP (EPACs)

S341 Phosphodiesterases, 3’,5’‐cyclic nucleotide (PDEs)

S344 Cytochrome P450

S344 CYP1 family

S345 CYP2 family: drug metabolising subset

S346 CYP2 family: physiological enzymes subset

S346 CYP3 family

S347 CYP4 family

S348 CYP5, CYP7 and CYP8 families

S349 CYP11, CYP17, CYP19, CYP20 and CYP21 families

S350 CYP24, CYP26 and CYP27 families

S350 CYP39, CYP46 and CYP51 families

S351 DNA topoisomerases

S351 E3 ubiquitin ligase components

S352 Endocannabinoid turnover

S353 N‐Acylethanolamine turnover

S354 2‐Acylglycerol ester turnover

S355 Eicosanoid turnover

S355 Cyclooxygenase

S356 Prostaglandin synthases

S358 Lipoxygenases

S359 Leukotriene and lipoxin metabolism

S359 GABA turnover

S361 Glycerophospholipid turnover

S361 Phosphoinositide‐specific phospholipase C

S363 Phospholipase A2

S364 Phosphatidylcholine‐specific phospholipase D

S365 Lipid phosphate phosphatases

S366 Phosphatidylinositol kinases

S368 Phosphatidylinositol phosphate kinases

S369 Haem oxygenase

S370 Hydrogen sulphide synthesis

S371 Hydrolases

S373 Inositol phosphate turnover

S373 Inositol 1,4,5‐trisphosphate 3‐kinases

S373 Inositol polyphosphate phosphatases

S374 Inositol monophosphatase

S374 Kinases (EC 2.7.x.x)

S375 Rho kinase

S375 Protein kinase C (PKC) family

S376 Alpha subfamily

S376 Delta subfamily

S377 Eta subfamily

S377 Iota subfamily

S378 FRAP subfamily

S378 Cyclin‐dependent kinase (CDK) family

S379 CDK4 subfamily

S379 GSK subfamily

S380 Polo‐like kinase (PLK) family

S381 STE7 family

S382 Abl family

S382 Ack family

S383 Janus kinase (JakA) family

S383 Src family

S384 Tec family

S385 RAF family

S385 Lanosterol biosynthesis pathway

S388 Nucleoside synthesis and metabolism

S389 Paraoxonase (PON) family

S390 Peptidases and proteinases

S390 Blood coagulation components

S391 A1: Pepsin

S391 A22: Presenilin

S392 C14: Caspase

S392 M1: Aminopeptidase N

S393 M2: Angiotensin‐converting enzymes (ACE and ACE2)

S393 M10: Matrix metallopeptidase

S394 M12: Astacin/Adamalysin

S394 M28: Aminopeptidase Y

S395 M19: Membrane dipeptidase

S395 S1: Chymotrypsin

S396 T1: Proteasome

S397 S8: Subtilisin

S397 S9: Prolyl oligopeptidase

S397 Peptidyl‐prolyl cis/trans isomerases

S399 Poly ADP‐ribose polymerases

S399 Prolyl hydroxylases

S400 Sphingosine 1‐phosphate turnover

S400 Sphingosine kinase

S402 Sphingosine 1‐phosphate phosphatase

S402 Sphingosine 1‐phosphate lyase

S403 Thyroid hormone turnover

S404 1.14.13.9 Kynurenine 3‐monooxygenase

S405 2.5.1.58 Protein farnesyltransferase

S405 3.5.3.15 Peptidyl arginine deiminases (PADI)

S406 3.6.5.2 Small monomeric GTPases

S406 RAS subfamily

S406 RAB subfamily

**S412 Transporters**

S414 ATP‐binding cassette transporter family

S415 ABCA subfamily

S416 ABCB subfamily

S417 ABCC subfamily

S418 ABCD subfamily of peroxisomal ABC transporters

S419 ABCG subfamily

S419 F‐type and V‐type ATPases

S420 F‐type ATPase

S420 V‐type ATPase

S420 P‐type ATPases

S421 P1B P‐type ATPases: Cu^+^‐ATPases

S421 P2A P‐type ATPases: Ca^2+^‐ATPases

S422 P2B P‐type ATPases: Ca^2+^‐ATPases

S422 Na^+^/K^+^‐ATPases

S422 H^+^/K^+^‐ATPases

S423 P4 P‐type ATPases: Phospholipid‐transporting ATPases

S423 P5 P‐type ATPases: Mn^2+^‐ATPases

S424 SLC superfamily of solute carriers

S425 SLC1 family of amino acid transporters

S425 Glutamate transporter subfamily

S427 Alanine/serine/cysteine transporter subfamily

S427 SLC2 family of hexose and sugar alcohol transporters

S428 Class I transporters

S428 Class II transporters

S429 Proton‐coupled inositol transporter

S430 SLC3 and SLC7 families of heteromeric amino acid transporters (HATs)

S430 SLC3 family

S430 SLC7 family

S432 SLC4 family of bicarbonate transporters

S432 Anion exchangers

S433 Sodium‐dependent HCO_3_^−^ transporters

S433 SLC5 family of sodium‐dependent glucose transporters

S434 Hexose transporter family

S435 Choline transporter

S436 Sodium iodide symporter, sodium‐dependent multivitamin transporter and sodium‐coupled monocarboxylate transporters

S437 Sodium myo‐inositol cotransporter transporters

S438 SLC6 neurotransmitter transporter family

S439 Monoamine transporter subfamily

S439 GABA transporter subfamily

S440 Glycine transporter subfamily

S442 Neutral amino acid transporter subfamily

S443 SLC8 family of sodium/calcium exchangers

S444 SLC9 family of sodium/hydrogen exchangers

S444 SLC10 family of sodium‐bile acid co‐transporters

S445 SLC11 family of proton‐coupled metal ion transporters

S446 SLC12 family of cation‐coupled chloride transporters

S448 SLC13 family of sodium‐dependent sulphate/carboxylate transporters

S449 SLC14 family of facilitative urea transporters

S450 SLC15 family of peptide transporters

S453 SLC16 family of monocarboxylate transporters

S454 SLC17 phosphate and organic anion transporter family

S454 Type I sodium‐phosphate co‐transporters

S455 Sialic acid transporter

S455 Vesicular glutamate transporters (VGLUTs)

S456 Vesicular nucleotide transporter

S456 SLC18 family of vesicular amine transporters

S457 SLC19 family of vitamin transporters

S458 SLC20 family of sodium‐dependent phosphate transporters

S459 SLC22 family of organic cation and anion transporters

S460 Organic cation transporters (OCT)

S461 Organic zwitterions/cation transporters (OCTN)

S461 Organic anion transporters (OATs)

S462 Urate transporter

S463 Atypical SLC22B subfamily

S464 SLC23 family of ascorbic acid transporters

S465 SLC24 family of sodium/potassium/calcium exchangers

S466 SLC25 family of mitochondrial transporters

S466 Mitochondrial di‐ and tri‐carboxylic acid transporter subfamily

S467 Mitochondrial amino acid transporter subfamily

S468 Mitochondrial phosphate transporters

S468 Mitochondrial nucleotide transporter subfamily

S469 Mitochondrial uncoupling proteins

S469 Miscellaneous SLC25 mitochondrial transporters

S470 SLC26 family of anion exchangers

S470 Selective sulphate transporters

S471 Chloride/bicarbonate exchangers

S471 Anion channels

S472 Other SLC26 anion exchangers

S472 SLC27 family of fatty acid transporters

S473 SLC28 and SLC29 families of nucleoside transporters

S474 SLC28 family

S475 SLC29 family

S476 SLC30 zinc transporter family

S477 SLC31 family of copper transporters

S478 SLC32 vesicular inhibitory amino acid transporter

S479 SLC33 acetylCoA transporter

S480 SLC34 family of sodium phosphate co‐transporters

S481 SLC35 family of nucleotide sugar transporters

S482 SLC36 family of proton‐coupled amino acid transporters

S483 SLC37 family of phosphosugar/phosphate exchangers

S484 SLC38 family of sodium‐dependent neutral amino acid transporters

S484 System A‐like transporters

S485 System N‐like transporters

S485 Orphan SLC38 transporters

S486 SLC39 family of metal ion transporters

S487 SLC40 iron transporter

S488 SLC41 family of divalent cation transporters

S489 SLC42 family of Rhesus glycoprotein ammonium transporters

S490 SLC43 family of large neutral amino acid transporters

S491 SLC44 choline transporter‐like family

S492 SLC45 family of putative sugar transporters

S493 SLC46 family of folate transporters

S494 SLC47 family of multidrug and toxin extrusion transporters

S495 SLC48 heme transporter

S495 SLC49 family of FLVCR‐related heme transporters

S496 SLC50 sugar transporter

S497 SLC51 family of steroid‐derived molecule transporters

S498 SLC52 family of riboflavin transporters

S499 SLC53 Phosphate carriers

S499 SLC54 Mitochondrial pyruvate carriers

S500 SLC55 Mitochondrial cation/proton exchangers

S500 SLC56 Sideroflexins

S501 SLC57 NiPA‐like magnesium transporter family

S501 SLC58 MagT‐like magnesium transporter family

S502 SLC59 Sodium‐dependent lysophosphatidylcholine symporter family

S502 SLC60 Glucose transporters

S503 SLC61 Molybdate transporter family

S503 SLC62 Pyrophosphate transporters

S504 SLC63 Sphingosine phosphate transporters

S504 SLC64 Golgi Ca^2+^/H^+^ exchangers

S505 SLC65 NPC‐type cholesterol transporters

S505 SLC66 Lysosomal amino acid transporters

S506 SLCO family of organic anion transporting polypeptides

## Introduction

In order to allow clarity and consistency in pharmacology, there is a need for a comprehensive organisation and presentation of the targets of drugs. This is the philosophy of the IUPHAR/BPS Guide to PHARMACOLOGY presented on the online free access database (http://www.guidetopharmacology.org/). This database is supported by the British Pharmacological Society (BPS), the International Union of Basic and Clinical Pharmacology (IUPHAR), the University of Edinburgh and previously the Wellcome Trust. Data included in the Guide to PHARMACOLOGY are derived in large part from interactions with the subcommittees of the Nomenclature amd Standards Committee of the International Union of Basic and Clinical Pharmacology (NC‐IUPHAR). A major influence on the development of the database was Tony Harmar (1951‐2014), who worked with a passion to establish the curators as a team of highly informed and informative individuals, with a focus on high‐quality data input, ensuring a suitably validated dataset. The Editors of the Concise Guide have compiled the individual records, in concert with the team of Curators, drawing on the expert knowledge of these latter subcommittees. The tables allow an indication of the status of the nomenclature for the group of targets listed, usually previously published in Pharmacological Reviews. In the absence of an established subcommittee, advice from several prominent, independent experts has generally been obtained to produce an authoritative consensus on nomenclature, which attempts to fit in within the general guidelines from NC‐IUPHAR. This current edition, the Concise Guide to PHARMACOLOGY 2021/22, is the latest snapshot of the database in print form, following on from the Concise Guide to PHARMACOLOGY 2019/20. It contains data drawn from the online database as a rapid overview of the major pharmacological targets. Thus, there are many fewer targets presented in the Concise Guide compared to the online database. The priority for inclusion in the Concise Guide is the presence of quantitative pharmacological data for human proteins. This means that often orphan family members are not presented in the Concise Guide, although structural information is available on the online database. The organisation of the data is tabular(where appropriate) with a standardised format, where possible on a single page, intended to aid understanding of, and comparison within, a particular target group. The Concise Guide is intended as an initial resource, with links to additional reviews and resources for greater depth and information. Pharmacological and structural data focus primarily on human gene products, wherever possible, with links to HGNC gene nomenclature and UniProt IDs. In a few cases, where data from human proteins are limited, data from other species are indicated. Pharmacological tools listed are prioritised on the basis of selectivity and availability. That is, agents(agonists, antagonists, inhibitors, activators, etc.) are included where they are both available (by donation or from commercial sources, now or in the near future) AND the most selective. The Concise Guide is divided into seven sections, which comprise pharmacological targets of similar structure/function. These are G protein‐coupled receptors, ion channels (combining previous records of ligand‐gated, voltage‐gated and other ion channels), catalytic receptors, nuclear hormone receptors, enzymes, transporters and other protein targets. We hope that the Concise Guide will provide for researchers, teachers and students a state‐of‐the art source of accurate, curated information on the background to their work that they will use in the Introductions to their Research Papers or Reviews, or in supporting their teaching and studies. We recommend that any citations to information in the Concise Guide are presented in the following format:

Alexander SPH *et al.* (2021). The Concise Guide to PHARMACOLOGY 2021/22: Overview. *Br J Pharmacol* 178: S1–S26.

In this overview are listed protein targets of pharmacological interest, which are not G protein‐coupled receptors, ion channels, nuclear hormone receptors, catalytic receptors, transporters or enzymes. For obvious reasons, we have included potential drug targets of the SARS‐CoV‐2 virus, despite the current limited pharmacological data.

## Acknowledgements

We are extremely grateful to the British Pharmacological Society and the International Union of Basic and Clinical Pharmacology, for financial support of the website and for advice from the NC‐IUPHAR subcommittees. We thank the University of Edinburgh, who host the www.guidetopharmacology.org website. Previously, IUPHAR and the Wellcome Trust (099156/Z/12/Z]) also supported the initiation and expansion of the database. We are also tremendously grateful to the long list of collaborators from NC‐IUPHAR subcommittees and beyond, who have assisted in the construction of the Concise Guide to PHARMACOLOGY 2021/22 and the online database www.GuideToPHARMACOLOGY.org.

## Conflict of interest

The authors state that there are no conflicts of interest to disclose.

## Family structure

– Abscisic acid receptor complex

S8 Adiponectin receptors

– Anti‐infective targets

– Antimalarial targets

– Other anti‐infective targets

S9 Aryl hydrocarbon receptor

– B‐cell lymphoma 2(Bcl‐2) protein family

– Bromodomain‐containing proteins

S10 Non‐enzymatic BRD containing proteins

– Butyrophilin and butyrophilin‐like proteins

S11 CD molecules

– Chaperone proteins

– Lipid binding chaperones

– Chitinase‐like proteins

– Chromatin‐interacting transcriptional repressors

S13 Methyllysine reader proteins

– Circadian clock proteins

– Claudins

– Cytolytic pore‐forming proteins

– EF‐hand domain containing proteins

S14 Fatty acid‐binding proteins

– Guanine nucleotide exchange factors (GEFs)

– Heat shock proteins

– Hypoxia‐inducible factors

– Immune checkpoint proteins

– Immunoglobulin C1‐set domain‐containing proteins

– Immunoglobulin C2‐set domain‐containing proteins

– Immunoglobulin like domain containing proteins

– Immunoglobulins

– Inhibitors of apoptosis (IAP) protein family

– Kelch‐like proteins

– Kinesins

– Leucine‐rich repeat proteins

– Lymphocyte antigens

– Mitochondrial‐associated proteins

– Myosin binding proteins

– Neuropilins and Plexins

– Non‐catalytic pattern recognition receptors

S16 Notch receptors

– Nuclear export proteins

– Pentraxins

S17 Regulators of G protein Signaling (RGS) proteins

S17 RZ family

S18 R4 family

S19 R7 family

S19 R12 family

– Repulsive guidance molecules

– Reticulons and associated proteins

– Ribosomal factors

– Sialic acid binding Ig like lectins

S20 Sigma receptors

– Signal regulatory proteins

– Tetraspanins

– Transcription factors

– Transcription factor regulators

–NF‐κB regulators

S21 Transthyretin

S22 Tubulins

– Tumour‐associated antigens

– WD repeat‐containing proteins

– Plasmodium multidrug resistance family

S23 SARS‐CoV‐2

S23 Structural proteins

S24 Polyproteins

S24 Proteases

S25 Nucleic acid turnover

S25 Other proteins

## Adiponectin receptors

### Overview

Adiponectin receptors (provisional nomenclature, ENSFM00500000270960) respond to the 30 kDa complement‐related protein hormone adiponectin (also known as *ADIPOQ* : adipocyte, C1q and collagen domain‐containing protein; ACRP30, adipose most abundant gene transcript 1; apM‐1; gelatin‐binding protein: Q15848) originally cloned from adipocytes [69]. Although sequence data suggest 7TM domains, immunological evidence indicates that, contrary to typical 7TM topology, the carboxyl terminus is extracellular, while the amino terminus is intracellular [136]. Signalling through these receptors appears to avoid G proteins; modelling based on the crystal structures of the adiponectin receptors suggested ceramidase acivity, which would make these the first in a new family of catalytic receptors [121].

### Further reading on Adiponectin receptors

Fisman EZ *et al.* (2014) Adiponectin: a manifold therapeutic target for metabolic syndrome, diabetes, and coronary disease? *Cardiovasc Diabetol* **13**: 103 [PMID:24957699]

Okada‐Iwabu M *et al.* (2018) Structure and function analysis of adiponectin receptors toward development of novel antidiabetic agents promoting healthy longevity. *Endocr J* **65**: 971‐977 [PMID:30282888]

Ruan H *et al.* (2016) Adiponectin signaling and function in insulin target tissues. *J Mol Cell Biol* **8**: 101‐9 [PMID:26993044]

Wang Y *et al.* (2017) Cardiovascular Adiponectin Resistance: The Critical Role of Adiponectin Receptor Modification. *Trends Endocrinol Metab* **28**: 519‐530 [PMID:28473178]

Zhao L *et al.* (2014) Adiponectin and insulin cross talk: the microvascular connection. *Trends Cardiovasc Med* **24**: 319‐24 [PMID:25220977]

**Table:** 

Nomenclature	Adipo1 receptor	Adipo2 receptor
HGNC, UniProt	*ADIPOR1* , Q96A54	*ADIPOR2* , Q86V24
Rank order of potency	globular adiponectin ( *ADIPOQ* , Q15848) > adiponectin ( *ADIPOQ* , Q15848)	globular adiponectin (ADIPOQ, Q15848) = adiponectin ( *ADIPOQ* , Q15848)

### Comments

T‐Cadherin ( *CDH13* , P55290) has also been suggested to be a receptor for (hexameric) adiponectin [47].

## Aryl hydrocarbon receptor

### Overview

The aryl hydrocarbon receptor, highly expressed in the liver and barrier organs, is resident in the cytoplasm bound to the chaperone heat shock protein hsp90. Upon agonist activation, the ligand:aryl hydrocarbon receptor complex migrates to the nucleus and binds the aryl hydrocarbon receptor nuclear translocator (ARNT, P27540, also known as HIF1β). The complex regulates transcription of selected genes through interaction with xenobiotic response elements (XRE). Among the genes regulated by the AHR/ARNT complex are cytochrome P450s, particularly CYP1A1, and the period circadian protein homolog 1 (PER1, O15534). The aryl hydrocarbon receptor is also capable of non‐genomic signalling.

### Further reading on Aryl hydrocarbon receptor

Bock KW. (2019) Aryl hydrocarbon receptor (AHR): From selected human target genes and crosstalk with transcription factors to multiple AHR functions. *Biochem Pharmacol* **168**: 65‐70 [PMID:31228464]

Bock KW. (2020) Aryl hydrocarbon receptor (AHR) functions: Balancing opposing processes including inflammatory reactions. *Biochem Pharmacol* **178**: 114093 [PMID:32535108]

Esser C *et al.* (2015) The aryl hydrocarbon receptor in barrier organ physiology, immunology, and toxicology. *Pharmacol Rev* **67**: 259‐79 [PMID:25657351]

Roman ÁC *et al.* (2018) The aryl hydrocarbon receptor in the crossroad of signalling networks with therapeutic value. *Pharmacol Ther* **185**: 50‐63 [PMID:29258844]

Rothhammer V *et al.* (2019) The aryl hydrocarbon receptor: an environmental sensor integrating immune responses in health and disease. *Nat Rev Immunol* **19**: 184‐197 [PMID:30718831]

Shi Y *et al.* (2020) The aryl hydrocarbon receptor: An environmental effector in the pathogenesis of fibrosis. *Pharmacol Res* **160**: 105180 [PMID:32877693]

**Table:** 

Nomenclature	Aryl hydrocarbon receptor
HGNC, UniProt	*AHR* , P35869
Agonists	indolo[3,2‐b]carbazole [12] – Mouse, tapinarof [110], indole‐3‐carbinol [12] – Mouse, TCDD
Antagonists	ezutromid (p*K*_d_ 7.3) [132]

## Non‐enzymatic BRD containing proteins

### Overview

Bromodomains bind proteins with acetylated lysine residues, such as histones, to regulate gene transcription. Listed herein are examples of bromodomain‐containing proteins for which sufficient pharmacology exists.

### Further reading on Non‐enzymatic BRD containing proteins

Fujisawa T *et al.* (2017) Functions of bromodomain‐containing proteins and their roles in homeostasis and cancer. *Nat Rev Mol Cell Biol* **18**: 246‐262 [PMID:28053347]

Myrianthopoulos V *et al.* (2019) From bench to bedside, via desktop. Recent advances in the application of cutting‐edge in silico tools in the research of drugs targeting bromodomain modules. *Biochem Pharmacol* **159**: 40‐51 [PMID:30414936]

Nicholas DA *et al.* (2017) BET bromodomain proteins and epigenetic regulation of inflammation: implications for type 2 diabetes and breast cancer. *Cell Mol Life Sci* **74**: 231‐243 [PMID:27491296]

Ramadoss M *et al.* (2018) Targeting the cancer epigenome: synergistic therapy with bromodomain inhibitors. *Drug Discov Today* **23**: 76‐89 [PMID:28943305]

Spriano F *et al.* (2020) Targeting BET bromodomain proteins in cancer: The example of lymphomas. *Pharmacol Ther* **215**: 107631 [PMID:32693114]

Tang P *et al.* (2021) Targeting Bromodomain and Extraterminal Proteins for Drug Discovery: From Current Progress to Technological Development. *J Med Chem* **64**: 2419‐2435 [PMID:33616410]

**Table:** 

Nomenclature	bromodomain adjacent to zinc finger domain 2A	bromodomain adjacent to zinc finger domain 2B	CREB binding protein	polybromo 1	SWI/SNF related, matrix associated, actin dependent regulator of chromatin, subfamily a, member 4
HGNC, UniProt	*BAZ2A* , Q9UIF9	*BAZ2B* , Q9UIF8	*CREBBP* , Q92793	*PBRM1* , Q86U86	*SMARCA4* , P51532
Selective inhibitors	GSK2801 (p*K*_d_ 6.6) [87]	GSK2801 (Binding) (p*K*_d_ 6.9) [87]	I‐CBP112 (p*K*_d_ 6.8) [88]	PFI‐3 (Binding) (p*K*_d_ 7.3) [101]	PFI‐3 (Binding) (p*K*_d_ 7.1) [101]

## CD molecules

### Overview

Cluster of differentiation refers to an attempt to catalogue systematically a series of over 300 cell‐surface proteins associated with immunotyping. Many members of the group have identified functions as enzymes (for example, see CD73 ecto‐5’‐nucleotidase) or receptors (for example, see CD41 integrin, alpha 2b subunit). Many CDs are targeted for therapeutic gain using antibodies for the treatment of proliferative disorders. A full listing of all the Clusters of Differentiation proteins is not possible in the Guide to PHARMACOLOGY; listed herein are selected members of the family targeted for therapeutic gain.

### Further reading on CD molecules

Bewersdorf JP *et al.* (2021) Immune checkpoint inhibition in myeloid malignancies: Moving beyond the PD‐1/PD‐L1 and CTLA‐4 pathways. *Blood Rev* **45**: 100709 [PMID:32487480]

Chi Z *et al.* (2021) Transcriptional and epigenetic regulation of PD‐1 expression. *Cell Mol Life Sci* **78**: 3239‐3246 [PMID:33738533]

Gabius HJ *et al.* (2015) The glycobiology of the CD system: a dictionary for translating marker designations into glycan/lectin structure and function. *Trends Biochem Sci* **40**: 360‐76 [PMID:25981696]

Huang MY *et al.* (2021) Combination therapy with PD‐1/PD‐L1 blockade in non‐small cell lung cancer: strategies and mechanisms. *Pharmacol Ther* **219**: 107694 [PMID:32980443]

Vosoughi T *et al.* (2019) CD markers variations in chronic lymphocytic leukemia: New insights into prognosis. *J Cell Physiol* **234**: 19420‐19439 [PMID:31049958]

**Table:** 

Nomenclature	CD2	CD3e	CD6	CD20 (membrane‐spanning 4‐domains, subfamily A, member 1)	CD33
Common abbreviation	–	–	–	–	SIGLEC3
HGNC, UniProt	*CD2* , P06729	*CD3E* , P07766	*CD6* , P30203	*MS4A1* , P11836	*CD33* , P20138
Selective inhibitors	alefacept [23, 74]	–	–	–	–
Antibodies	–	catumaxomab (Binding) [63], muromonab‐CD3 (Binding) [32], otelixizumab (Binding) [14]	–	ofatumumab (Binding) (p*K*_d_ 9.9) [58], rituximab (Binding) (p*K*_d_ 8.5) [113], ibritumomab tiuxetan (Binding), obinutuzumab (Binding) [3, 90], tositumomab (Binding)	lintuzumab (Binding) (p*K*_d_∼10) [16], gemtuzumab ozogamicin (Binding) [10]

**Table:** 

Nomenclature	CD52	CD80	CD86	cytotoxic T‐lymphocyte‐associated protein 4 (CD152)	programmed cell death 1 (CD279)	CD300a
Common abbreviation	–	–	–	CTLA‐4	PD‐1	–
HGNC, UniProt	*CD52* , P31358	*CD80* , P33681	*CD86* , P42081	*CTLA4* , P16410	*PDCD1* , Q15116	*CD300A* , Q9UGN4
Endogenous ligands	–	–	–	–	programmed cell death 1 ligand 1 (CD274, Q9NZQ7) (Binding)	–
Selective inhibitors	–	abatacept (p*K*_d_∼7.9) [64, 125]	abatacept (p*K*_d_∼7.9) [64, 125], belatacept [57]	–	–	–
Antibodies	alemtuzumab (Binding) [30, 89]	–	–	ipilimumab (Binding) (p*K*_d_>9) [33], tremelimumab (Binding) (p*K*_d_ 8.9) [35]	pembrolizumab (Binding) (p*K*_d_∼10) [17], nivolumab (Binding) (p*K*_d_ 9.1) [38, 54, 49]	–

### Comments

The endogenous ligands for human PD‐1 are programmed cell death 1 ligand 1 (PD‐L1 aka CD274 (CD274, Q9NZQ7)) and programmed cell death 1 ligand 2 (PD‐L2; PDCD1LG2). These ligands are cell surface peptides, normally involved in immune system regulation. Expression of PD‐1 by cancer cells induces immune tolerance and evasion of immune system attack. Anti‐PD‐1 monoclonal antibodies are used to induce immune checkpoint blockade as a therapeutic intervention in cancer, effectively re‐establishing immune vigilance. Pembrolizumab was the first anti‐PD‐1 antibody to be approved by the US FDA.

## Methyllysine reader proteins

### Overview

Methyllysine reader proteins bind to methylated proteins, such as histones, allowing regulation of gene expression.

### Further reading on Methyllysine reader proteins

Daskalaki MG *et al.* (2018) Histone methylation and acetylation in macrophages as a mechanism for regulation of inflammatory responses. *J Cell Physiol* **233**: 6495‐6507 [PMID:29574768]

Furuya K *et al.* (2019) Epigenetic interplays between DNA demethylation and histone methylation for protecting oncogenesis. *J Biochem* **165**: 297‐299 [PMID:30605533]

Levy D. (2019) Lysine methylation signaling of non‐histone proteins in the nucleus. *Cell Mol Life Sci* **76**: 2873‐2883 [PMID:31123776]

Li J *et al.* (2019) Understanding histone H3 lysine 36 methylation and its deregulation in disease. *Cell Mol Life Sci* **76**: 2899‐2916 [PMID:31147750]

Shafabakhsh R *et al.* (2019) Role of histone modification and DNA methylation in signaling pathways involved in diabetic retinopathy. *J Cell Physiol* **234**: 7839‐7846 [PMID:30515789]

**Table:** 

Nomenclature	L3MBTL histone methyl‐lysine binding protein 3
HGNC, UniProt	*L3MBTL3* , Q96JM7
Selective agonists	UNC1215 [50]

## Fatty acid‐binding proteins

### Overview

Fatty acid‐binding proteins are low molecular weight (100‐130 aa) chaperones for long chain fatty acids, fatty acyl CoA esters, eicosanoids, retinols, retinoic acids and related metabolites and are usually regarded as being responsible for allowing the otherwise hydrophobic ligands to be mobile in aqueous media. These binding proteins may perform functions extracellularly (e.g. in plasma) or transport these agents; to the nucleus to interact with nuclear receptors (principally PPARs and retinoic acid receptors [99]) or for interaction with metabolic enzymes. Although sequence homology is limited, crystallographic studies suggest conserved 3D structures across the group of binding proteins.

### Further reading on Fatty acid‐binding proteins

Gajda AM *et al.* (2015) Enterocyte fatty acid‐binding proteins (FABPs): different functions of liver and intestinal FABPs in the intestine. *Prostaglandins Leukot Essent Fatty Acids* **93**: 9‐16 [PMID:25458898]

Glatz JF. (2015) Lipids and lipid binding proteins: a perfect match. *Prostaglandins Leukot Essent Fatty Acids* **93**: 45‐9 [PMID:25154384]

Hotamisligil GS *et al.* (2015) Metabolic functions of FABPs–mechanisms and therapeutic implications. *Nat Rev Endocrinol* **11**: 592‐605 [PMID:26260145]

Matsumata M *et al.* (2016) Fatty acid binding proteins and the nervous system: Their impact on mental conditions. *Neurosci Res* **102**: 47‐55 [PMID:25205626]

Nguyen HC *et al.* (2020) Role of the Fatty Acid Binding Proteins in Cardiovascular Diseases: A Systematic Review. *J Clin Med* **9**: [PMID:33105856]

Osumi T *et al.* (2016) Heart lipid droplets and lipid droplet‐binding proteins: Biochemistry, physiology, and pathology. *Exp Cell Res* **340**: 198‐204 [PMID:26524506]

**Table:** 

Nomenclature	fatty acid binding protein 1	fatty acid binding protein 2	fatty acid binding protein 3	fatty acid binding protein 4	fatty acid binding protein 5
HGNC, UniProt	FABP1, P07148	FABP2, P12104	FABP3, P05413	FABP4, P15090	FABP5, Q01469
Rank order of potency	stearic acid, oleic acid> palmitic acid, linoleic acid> arachidonic acid, α‐linolenic acid [91]	stearic acid> palmitic acid, oleic acid> linoleic acid> arachidonic acid, α‐linolenic acid [91]	stearic acid, oleic acid, palmitic acid> linoleic acid, α‐linolenic acid, arachidonic acid [91]	oleic acid, palmitic acid, stearic acid, linoleic acid> α‐linolenic acid, arachidonic acid [91]	–
Inhibitors	fenofibrate (pK_i_ 7.6) [18] – Rat, fenofibric acid (pK_i_ 6.5) [18] – Rat, HTS01037 (pK_i_ 5.1) [42] – Mouse	–	–	–	compound 13 (pK_i_ 8.7) [118]
Selective inhibitors	–	–	–	HM50316 (pK_i_>9) [66]	–
Comments	A broader substrate specificity than other FABPs, binding two fatty acids per protein [123].	Crystal structure of the rat FABP2 [95].	Crystal structure of the human FABP3 [137].	–	Crystal structure of the human FABP5 [44].

**Table:** 

Nomenclature	fatty acid binding protein 6	fatty acid binding protein 7	peripheral myelin protein 2	fatty acid binding protein 9	fatty acid binding protein 12
HGNC, UniProt	FABP6, P51161	FABP7, O15540	PMP2, P02689	FABP9, Q0Z7S8	FABP12, A6NFH5
Comments	Able to transport bile acids [142].	Crystal structure of the human FABP7 [9].	In silico modelling suggests that PMP2/FABP8 can bind both fatty acids and cholesterol [70].	–	–

**Table:** 

Nomenclature	retinol binding protein 1	retinol binding protein 2	retinol binding protein 3	retinol binding protein 4	retinol binding protein 5	retinol binding protein 7
HGNC, UniProt	RBP1, P09455	RBP2, P50120	RBP3, P10745	RBP4, P02753	RBP5, P82980	RBP7, Q96R05
Rank order of potency	–	stearic acid> palmitic acid, oleic acid, linoleic acid, α‐linolenic acid, arachidonic acid [92]	–	–	–	–
Inhibitors	–	–	–	A1120 (pIC_50_ 7.8) [128]	–	–

**Table:** 

Nomenclature	retinaldehyde binding protein 1	cellular retinoic acid binding protein 1	cellular retinoic acid binding protein 2
HGNC, UniProt	RLBP1, P12271	CRABP1, P29762	CRABP2, P29373
Rank order of potency	11‐cis‐retinal, 11‐cis‐retinol> 9‐cis‐retinal, 13‐cis‐retinal, 13‐cis‐retinol, all‐trans‐retinal, retinol [22]	tretinoin> alitretinoin stearic acid> palmitic acid, oleic acid, linoleic acid, α‐linolenic acid, arachidonic acid [92]	–

### Comments

Although not tested at all FABPs, BMS309403 exhibits high affinity for FABP4 (pIC50 ˜8.8) compared to FABP3 or FABP5 (pIC50<6.6) [27, 118]. HTS01037 is reported to interfere with FABP4 action [42]. Ibuprofen displays some selectivity for FABP4 (pIC_50_ 5.5) relative to FABP3 (pIC_50_ 3.5) and FABP5 (pIC_50_ 3.8) [68]. Fenofibric acid displays some selectivity for FABP5 (pIC_50_ 5.5) relative to FABP3 (pIC_50_ 4.5) and FABP4 (pIC_50_ 4.6) [68]. Multiple pseudogenes for the FABPs have been identified in the human genome.

## Notch receptors

### Overview

Aberrant Notch signalling is implicated in a number of human cancers [59, 80, 108, 126], and there is intense pharmaceutical activity being directed towards achieving clinically effective Notch pathway inhibition [24, 75].

### Further reading on Notch receptors

Fabbro D *et al.* (2020) Notch Inhibition in Cancer: Challenges and Opportunities. *Chimia (Aarau)* **74**: 779‐783 [PMID:33115560]

Moore G *et al.* (2020) Top Notch Targeting Strategies in Cancer: A Detailed Overview of Recent Insights and Current Perspectives. *Cells* **9**: [PMID:32575680]

Palmer WH *et al.* (2015) Ligand‐Independent Mechanisms of Notch Activity. *Trends Cell Biol* **25**: 697‐707 [PMID:26437585]

Previs RA *et al.* (2015) Molecular pathways: translational and therapeutic implications of the Notch signaling pathway in cancer. *Clin Cancer Res* **21**: 955‐61 [PMID:25388163]

Takebe N *et al.* (2015) Targeting Notch, Hedgehog, and Wnt pathways in cancer stem cells: clinical update. *Nat Rev Clin Oncol* **12**: 445‐64 [PMID:25850553]

**Table:** 

Nomenclature	notch receptor 1	notch receptor 2	notch receptor 3	notch receptor 4
HGNC, UniProt	NOTCH1, P46531	NOTCH2, Q04721	NOTCH3, Q9UM47	NOTCH4, Q99466
Inhibitors	IMR‐1 (Binding) (p*K*_d_ 5) [8]	–	–	–
Antibodies	brontictuzumab (Binding) (p*K*_d_ 8.4) [30]	tarextumab (Binding) (p*K*_d_>10) [31]	tarextumab (Binding) (p*K*_d_ 9.9) [31]	–
Comments	Various types of activating and inactivating NOTCH1 mutations have been reported to be associated with human diseases, for example: aortic valve disease [29, 73], Adams‐Oliver syndrome 5 [114], T‐cell acute lymphoblastic leukemia (T‐ALL) [130], chronic lymphocytic leukemia (CLL) [89] and head and neck squamous cell carcinoma [1, 115].	–	–	Notch receptor 4 is a potential therapeutic molecular target for triple‐negative breast cancer [60, 77].

## Regulators of G protein Signaling (RGS) proteins

### Overview

Regulator of G protein Signaling, or RGS, proteins serve an important regulatory role in signaling mediated by G protein‐coupled receptors (GPCRs). They all share a common RGS domain that directly interacts with active, GTP‐bound Gα subunits of heterotrimeric G proteins. RGS proteins stabilize the transition state for GTP hydrolysis on Gα and thus induce a conformational change in the Gα subunit that accelerates GTP hydrolysis, thereby effectively turning off signaling cascades mediated by GPCRs. This GTPase accelerating protein (GAP) activity is the canonical mechanism of action for RGS proteins, although many also possess additional functions and domains. RGS proteins are divided into four families, R4, R7, R12 and RZ based on sequence homology, domain structure as well as specificity towards Gα subunits. For reviews on RGS proteins and their potential as therapeutic targets, see e.g. [5, 45, 79, 93, 105, 106, 107, 138, 140].

### Further reading on Regulators of G protein Signaling (RGS) proteins

Alqinyah M *et al.* (2018) Regulating the regulators: Epigenetic, transcriptional, and post‐translational regulation of RGS proteins. *Cell Signal* **42**: 77‐87 [PMID:29042285]

Fuentes N *et al.* (2021) RGS proteins, GRKs, and beta‐arrestins modulate G protein‐mediated signaling pathways in asthma. *Pharmacol Ther* **223**: 107818 [PMID:33600853]

Neubig RR *et al.* (2002) Regulators of G‐protein signalling as new central nervous system drug targets. *Nat Rev Drug Discov* **1**: 187‐97 [PMID:12120503]

Sethakorn N *et al.* (2010) Non‐canonical functions of RGS proteins. *Cell Signal* **22**: 1274‐81 [PMID:20363320]

Sjögren B. (2017) The evolution of regulators of G protein signalling proteins as drug targets ‐ 20 years in the making: IUPHAR Review 21. *Br J Pharmacol* **174**: 427‐437 [PMID:28098342]

Sjögren B *et al.* (2010) Thinking outside of the "RGS box": new approaches to therapeutic targeting of regulators of G protein signaling. *Mol Pharmacol* **78**: 550‐7 [PMID:20664002]

## RZ family

### Overview

The RZ family of RGS proteins is less well characterized than the other families. It consists of, RGS17(also known as RGSZ2), RGS19 (also known as GAIP) and RGS20 (with several splice variants including RGSZ1 and Ret‐RGS). All members contain an N‐terminal cysteine string motif [62] which is a site of palmitoylation and could serve functions in membrane targeting, protein stability or aid protein‐protein interactions [2, 62]. However, the function in the case of RZ family RGS proteins is not yet fully understood. Members of the RZ family of RGS proteins are the only RGS proteins that have selective GAP activity for Gα_z_, a function that resulted in the name of the family [31, 71, 127, 134]. However, the members of the RZ family are able to also GAP Gα_i/o_ members with varying selectivity.

**Table:** 

Nomenclature	regulator of G‐protein signaling 17	regulator of G‐protein signaling 19	regulator of G‐protein signaling 20
Common abbreviation	RGS17	RGS19	RGS20
HGNC, UniProt	RGS17, Q9UGC6	RGS19, P49795	RGS20, O76081

## R4 family

### Overview

The R4 family of RGS proteins is the largest family of RGS proteins with 10 members. Each of the R4 family members contain only small N‐ and C‐termini apart from the RGS domain. The N‐terminal amphipathic helix present in most R4 family members serves an important function in membrane association and can directly bind phospholipids. In contrast to the RGS domain, which is well conserved among members of the R4 family of RGS proteins, the N‐ and C‐termini vary, enabling specificity of non‐GAP functions. Despite the non‐complex structure of these proteins, several R4 family RGS proteins have been shown to possess additional functions apart from acting as GAPs at activated Gα subunits [11, 96].

### Further reading on R4 family

Xie Z *et al.* (2016) R4 Regulator of G Protein Signaling (RGS) Proteins in Inflammation and Immunity. *AAPS J* **18**: 294‐304 [PMID:26597290]

**Table:** 

Nomenclature	regulator of G‐protein signaling 1	regulator of G‐protein signaling 2	regulator of G‐protein signaling 3	regulator of G‐protein signaling 4
Common abbreviation	RGS1	RGS2	RGS3	RGS4
HGNC, UniProt	RGS1, Q08116	RGS2, P41220	RGS3, P49796	RGS4, P49798
Selective inhibitors	–	–	–	RGS4 inhibitor 11b (pIC_50_ 7.8) [124], CCG‐50014 (pIC_50_ 7.5) [13, 124], RGS4 inhibitor 13 (pIC_50_ 7.3) [124]

**Table:** 

Nomenclature	regulator of G‐protein signaling 5	regulator of G‐protein signaling 8	regulator of G‐protein signaling 13	regulator of G‐protein signaling 16	regulator of G‐protein signaling 18	regulator of G‐protein signaling 21
Common abbreviation	RGS5	RGS8	RGS13	RGS16	RGS18	RGS21
HGNC, UniProt	RGS5, O15539	RGS8, P57771	RGS13, O14921	RGS16, O15492	RGS18, Q9NS28	RGS21, Q2M5E4

## R7 family

### Overview

The members of the R7 family of RGS proteins [6] are more complex structures than the R4 family and are closely related to the C. elegans homologues EGL‐10 and EAT‐16 that were identified in the early stage of RGS protein research [36, 55]. Apart from the RGS domain, several additional domains are present in these proteins that mediate protein‐protein interactions, sub‐cellular localization and protein stability. All R7 family members form obligatory dimers with Gβ5 through the G‐γ like (GGL) domain and the disheveled‐EGL10‐Pleckstrin homology (DEP) domain [109]. The DEP and DEP helical extension domain interact with R7 binding protein (R7BP) or RGS9 anchoring protein (R9AP; in retina) that serves as a plasma membrane anchoring mechanism [41, 51].

**Table:** 

Nomenclature	regulator of G‐protein signaling 6	regulator of G‐protein signaling 7	regulator of G‐protein signaling 9	regulator of G‐protein signaling 11
Common abbreviation	RGS6	RGS7	RGS9	RGS11
HGNC, UniProt	RGS6, P49758	RGS7, P49802	RGS9, O75916	RGS11, O94810

## R12 family

### Overview

The R12 family consisting of RGS10, 12 and 14. RGS12 and 14 are large proteins with additional domains that can participate in protein‐protein interactions and other functions. In contrast, RGS10 is a small protein consisting of the RGS domain and small N‐ and C‐termini, similar to members of the R4 family. However, the sequence homology the RGS10 RGS domain clearly places it in the R12 family [58]. The Gα_i/o_‐Loco(GoLoco) motif in RGS12 and 14 has GDI activity (for Guanine nucleotide Dissociation Inhibitor) towards Gα_i1_, Gα_i2_ and Gα_i3_ [53, 105]. Through this activity RGS12 and RGS14 can inhibit G protein signaling both by accelerating GTP hydrolysis and by preventing G protein activation. Splice variants of RGS12 and RGS14 also contain membrane targeting and protein‐protein interaction domains [97, 111, 112].

**Table:** 

Nomenclature	regulator of G‐protein signaling 10	regulator of G‐protein signaling 12	regulator of G‐protein signaling 14
Common abbreviation	RGS10	RGS12	RGS14
HGNC, UniProt	RGS10, O43665	RGS12, O14924	RGS14, O43566

## Sigma receptors

### Overview

Although termed ’receptors’, the evidence for coupling through conventional signalling pathways is lacking. Initially described as a subtype of opioid receptors, there is only a modest pharmacological overlap and no structural convergence with the G protein‐coupled receptors; the crystal structure of the sigma1 receptor [98] suggests a trimeric structure of a single short transmembrane domain traversing the endoplasmic reticulum membrane, with the bulk of the protein facing the cytosol. A wide range of compounds, ranging from psychoactive agents to antihistamines, have been observed to bind to these sites.

### Further reading on Sigma receptors

Chu UB *et al.* (2016) Biochemical Pharmacology of the Sigma‐1 Receptor. *Mol Pharmacol* **89**: 142‐53 [PMID:26560551]

Herrando‐Grabulosa M *et al.* (2021) Sigma 1 receptor as a therapeutic target for amyotrophic lateral sclerosis. *Br J Pharmacol* **178**: 1336‐1352 [PMID:32761823]

Sambo DO *et al.* (2018) The sigma‐1 receptor as a regulator of dopamine neurotransmission: A potential therapeutic target for methamphetamine addiction. *Pharmacol Ther* **186**: 152‐167 [PMID:29360540]

Schmidt HR *et al.* (2019) The Molecular Function ofσ Receptors: Past, Present, and Future. *Trends Pharmacol Sci* **40**: 636‐654 [PMID:31387763]

Su TP *et al.* (2016) The Sigma‐1 Receptor as a Pluripotent Modulator in Living Systems. *Trends Pharmacol Sci* **37**: 262‐278 [PMID:26869505]

Vavers E *et al.* (2019) Allosteric Modulators of Sigma‐1 Receptor: A Review. *Front Pharmacol* **10**: 223 [PMID:30941035]

**Table:** 

Nomenclature	sigma non‐opioid intracellular receptor 1	σ2
HGNC, UniProt	SIGMAR1, Q99720	TMEM97, Q5BJF2
Agonists	–	1,3‐ditolylguanidine [61] – Guinea pig
Selective agonists	PRE‐084 [117], (+)‐SKF 10.047	–
Antagonists	–	SM 21 (pIC_50_ 7.2) [67]
Selective antagonists	NE‐100 (pIC_50_ 8.4) [81], BD‐1047 (pIC_50_ 7.4) [72]	–
Labelled ligands	[^3^H]pentazocine (Agonist)	[^3^H]‐di‐o‐tolylguanidine (Agonist)
Comments	–	The sigma2 receptor has been reported to be TMEM97 [4], a 4TM protein partner of NPC1, the Niemann‐Pick C1 protein, a 13TM cholesterol‐binding protein.

### Comments

(‐)‐pentazocine also shows activity at opioid receptors. The sigma2 receptor has recently been reported to be TMEM97 [4], a 4TM protein partner of NPC1, the Niemann‐Pick C1 protein, a 13TM cholesterol‐binding protein.

## Transthyretin

### Overview

Transthyretin (TTR) is a homo‐tetrameric protein which transports thyroxine in the plasma and cerebrospinal fluid and retinol (vitamin A) in the plasma. Many disease causing mutations in the protein have been reported, many of which cause complex dissociation and protein mis‐assembly and deposition of toxic aggregates amyloid fibril formation [84]. These amyloidogenic mutants are linked to the development of pathological amyloidoses, including familial amyloid polyneuropathy (FAP) [7, 20], familial amyloid cardiomyopathy(FAC) [49], amyloidotic vitreous opacities, carpal tunnel syndrome [76] and others. In old age, non‐mutated TTR can also form pathological amyloid fibrils [131]. Pharmacological intervention to reduce or prevent TTR dissociation is being pursued as a therapeutic strategy. To date one small molecule kinetic stabilising molecule (tafamidis) has been approved for FAP, and is being evaluated in clinical trials for other TTR amyloidoses.

### Further reading on Transthyretin

Adams D *et al.* (2019) Hereditary transthyretin amyloidosis: a model of medical progress for a fatal disease. *Nat Rev Neurol* **15**: 387‐404 [PMID:31209302]

Bezerra F *et al.* (2020) Modulation of the Mechanisms Driving Transthyretin Amyloidosis. *Front Mol Neurosci* **13**: 592644 [PMID:33362465]

Dohrn MF *et al.* (2020) Targeting transthyretin ‐ Mechanism‐based treatment approaches and future perspectives in hereditary amyloidosis. *J Neurochem* [PMID:33155274]

Galant NJ *et al.* (2017) Transthyretin amyloidosis: an under‐recognized neuropathy and cardiomyopathy. *Clin Sci* **131**: 395‐409 [PMID:28213611]

Griffin JM *et al.* (2019) Transthyretin cardiac amyloidosis: A treatable form of heart failure with a preserved ejection fraction. *Trends Cardiovasc Med* [PMID:31889610]

**Table:** 

Nomenclature	transthyretin
Common abbreviation	TTR
HGNC, UniProt	TTR, P02766
Inhibitors	tafamidis (p*K*_d_ 8.7) [15]

### Comments

Excess production and accumulation of TTR causes hereditary transthyretin‐mediated amyloidosis. Two novel drugs are now approved to combat this disease: inotersen(Tegsedi^*®*^) [52] and patisiran(Onpattro^*®*^) [46]. Both of these drugs act to reduce the amount of TTR protein (both wild type and mutant) produced in the liver, but by slightly different mechanisms. Inotersen is an antisense oligonucleotide inhibitor of TTR synthesis, whereas patisiran is a double‐stranded small interfering RNA (which targets a conserved sequence in the 3’ UTR of mutant and wild‐type TTR mRNA). Inotersen is administered subcutaneously, and patisiran is delivered by intravenous infusion in a lipid nanoparticle formulation.

## Tubulins

### Overview

Tubulins are a family of intracellular proteins most commonly associated with microtubules, part of the cytoskeleton. They are exploited for therapeutic gain in cancer chemotherapy as targets for agents derived from a variety of natural products: taxanes, colchicine and vinca alkaloids. These are thought to act primarily throughβ‐tubulin, thereby interfering with the normal processes of tubulin polymer formation and disassembly.

### Further reading on Tubulins

Arnst KE *et al.* (2019) Current advances of tubulin inhibitors as dual acting small molecules for cancer therapy. *Med Res Rev* **39**: 1398‐1426 [PMID:30746734]

Boiarska Z *et al.* (2021) Microtubule‐targeting agents and neurodegeneration. *Drug Discov Today* **26**: 604‐615 [PMID:33279455]

Eshun‐Wilson L *et al.* (2019) Effects of α‐tubulin acetylation on microtubule structure and stability. *Proc Natl Acad Sci USA* **116**: 10366‐10371 [PMID:31072936]

Gadadhar S *et al.* (2017) The tubulin code at a glance. *J Cell Sci* **130**: 1347‐1353 [PMID:28325758]

Magiera MM *et al.* (2018) Tubulin Posttranslational Modifications and Emerging Links to Human Disease. *Cell* **173**: 1323‐1327 [PMID:29856952]

Penna LS *et al.* (2017) Anti‐mitotic agents: Are they emerging molecules for cancer treatment? *Pharmacol Ther* **173**: 67‐82 [PMID:28174095]

**Table:** 

Nomenclature	tubulin alpha 1a	tubulin alpha 4a	tubulin beta class I	tubulin beta 3 class III	tubulin beta 4B class IVb	tubulin beta 8 class VIII
HGNC, UniProt	TUBA1A, Q71U36	TUBA4A, P68366	TUBB, P07437	TUBB3, Q13509	TUBB4B, P68371	TUBB8, Q3ZCM7
Inhibitors	–	–	vinblastine (pIC_50_ 9), eribulin (pIC_50_ 8.2) [78], paclitaxel (Mitotic cell cycle arrest in A431 cells) (pEC_50_ 8.1) [83], colchicine (pIC_50_ 8) [19], cabazitaxel, docetaxel, ixabepilone, vincristine	combretastatin A4 (pIC_50_ 8.2) [28]	–	–

## SARS‐CoV‐2

Coronaviruses are large, often spherical, enveloped, single‐stranded positive‐sense RNA viruses, ranging in size from 80–220 nm. Their genomes and protein structures are highly conserved. Three coronaviruses have emerged over the last 20 years as serious human pathogens: SARS‐CoV was identified as the causative agent in an outbreak in 2002–2003, Middle East respiratory syndrome (MERS) CoV emerged in 2012 and the novel coronavirus SARS‐CoV‐2 emerged in 2019–2020. SARS‐CoV‐2 is the virus responsible for the infectious disease termed COVID‐19 (WHO Technical Guidance 2020).

### Further reading on Tubulins

Alexander SPH *et al.* (2020) A rational roadmap for SARS‐CoV‐2/COVID‐19 pharmacotherapeutic research and development: IUPHAR Review 29. *Br J Pharmacol*, 177 (21): 4942‐4966. [PMID:32358833]

Cannalire R *et al.* (2020) Targeting SARS‐CoV‐2 Proteases and Polymerase for COVID‐19 Treatment: State of the Art and Future Opportunities. *J Med Chem*, [Epub ahead of print]. [PMID:33186044]

Cui J *et al.* (2019) Origin and evolution of pathogenic coronaviruses. *Nat Rev Microbiol*, 17 (3): 181‐192. [PMID:30531947]

Desforges M *et al.* (2019) Human Coronaviruses and Other Respiratory Viruses: Underestimated Opportunistic Pathogens of the Central Nervous System?. *Viruses*, 12 (1). [PMID:31861926]

Song Z *et al.* (2019) From SARS to MERS, Thrusting Coronaviruses into the Spotlight. *Viruses*, 11 (1). [PMID:30646565]

Zumla A *et al.* (2016) Coronaviruses ‐ drug discovery and therapeutic options. *Nat Rev Drug Discov*, 15(5): 327‐47. [PMID:26868298]

## Structural proteins

### Overview

The virus particle has four structural proteins. The envelope, membrane and spike proteins are on the viral surface, while the polybasic nucleoprotein enables the tight coiling of the viral RNA.

**Table:** 

Nomenclature	Envelope protein	Membrane glycoprotein	Nucleoprotein	Spike glycorprotein
Other names	envelope small membrane protein, orf4	Membrane protein	Nucleocapsid protein	Spike protein
UniProt	P0DTC4	P0DTC5	P0DTC9	P0DTC2
Function	By similarity to other coronavirus E proteins, SARS‐CoV‐2 E is predicted to constitute a single transmembrane (potentially homopentameric) ion channel with selectivity for monovalent cations over monovalent anions [85, 119, 133, 139]	The membrane glycoprotein (M) is usually regarded as the most abundant protein in the coronavirus envelope. By similarity with other coronavirus M proteins it is predicted to be essential for initiating assembly of the viral envelope components [94]	The coronavirus nucleocapsid phosphoprotein (N, or nucleoprotein) is highly basic and binds the viral RNA as a dimeric entity [25] into nucleocapsids which protect the viral genome, while also providing access for replication when required	The spike protein extends from the viral surface and binds to the host cell surface enzyme ACE2 to facilitate viral entry into the cell

## Polyproteins

### Overview

The viral RNA encodes two overlapping polyproteins which are cleaved autocatalytically by intrinsic proteases (see below).

**Table:** 

Nomenclature	Replicase polyprotein 1a	Replicase polyprotein 1ab
Other names	Polyprotein 1a	Polyprotein 1ab
UniProt	P0DTC1	P0DTD1
Function	The replicase polyprotein 1a (pp1a) encodes a set of 11 smaller proteins, including two proteases that are responsible for cleaving the polyprotein chain into its component parts	The replicase polyprotein 1ab (pp1ab) encodes a set of 16 smaller proteins (5 more than pp1a)

### Comment

The component proteins are non‐structural and are involved in the transcription and replication of viral proteins and RNA.

## Proteases

**Table:** 

Nomenclature	3C‐like (main) protease	Papain‐like protease
Other names	3c‐like proteinase, SARS‐CoV‐2 Mpro, Chain A, 3c‐like Proteinase, 3CL protease, Mpro, nsp5	non‐structural protein 3, NS3, nsp3, PL‐PRO
UniProt	P0C6U8	P0DTC1
EC number	3.4.22.69	3.4.22.46
Function	The 3C‐like protease cleaves the two polyproteins encoded by the SARS‐CoV‐2 genome (pp1a and pp1ab) into a range of non‐structural proteins (nsp1‐11 from pp1a; nsp1‐16 from pp1ab). As these component proteins play crucial roles in viral replication, the 3C‐like protease is considered to be a good molecular target for drug development. Small molecule 3C‐like protease inhibitors would be predicted to reduce viral replication [33, 85]	The papain‐like protease is a domain within coronavirus Nsp3. Its proteolytic activity cleaves three sites in the viral replicase polyprotein (recognition consensus sequence LXGG*↓*XX) to release the three non‐structural proteins Nsp1, Nsp2, and Nsp3 [40]. It has additional non‐proteolytic functions as part of the multicomponent replicase‐transcriptase complex [103]

## Nucleic acid turnover

**Table:** 

Nomenclature	Non‐structural protein 8	RNA‐dependent RNA polymerase
Other names	Nsp8	non‐structural protein 12, nsp12
UniProt	P0DTC8	P0DTD1
Function	Coronavirus nsp8 proteins form a hexadecameric complex with nsp7 proteins (8 subunits of each) [48, 122]. This complex may participate in viral replication by acting as a primase for de novo initiation of RNA synthesis	The conservation of RdRP catalytic domain between different RNA viruses endows inhibitors that were designed against other viral pathogens with activity against the SARS coronaviruses. Viral RdRP is the molecular target of nucleotide‐based broad‐spectrum antiviral compounds like remdesivir, tenofovir and ribavirin [33, 129, 141]

## Other proteins

**Table:** 

Nomenclature	Protein 3a	Protein 7a	Protein 9b	Non‐structural protein 6	Non‐structural protein 7b
Other names	Orf3a	Orf7a	Orf9b, Accessory protein 9b, ORF‐9b	Nsp6	Accessory protein 7b, nsp7b
UniProt	P0DTC3	P0DTC7	P0DTD2	P0DTC6	P0DTD8
Function	Protein 3a is a transmembrane pore‐forming viral protein (viroporin) with potassium ion channel activity	The main function of the SARS‐CoV protein 7a appears to be disruption of the host cell cycle and induction of caspase‐dependent apoptosis [120]. By homology SARS‐CoV‐2 protein 7a is likely to produce the same effect	SARS‐CoV protein 9b is a virion‐associated accessory protein [120] that acts to block the host’s ability to mount an antiviral IFN‐induced innate immune response [87]. By homology, 9b from SARS‐CoV‐2 would be predicted to exhibit a similar function	Coronavirus nsp6 proteins limit autophagosome expansion [21]. This mechanism may favour coronavirus infection by damaging autophagosome‐mediated delivery of viral components to lysosomes for degradation	Protein 7b is a coronavirus accessory protein. Experimental evidence suggests that SARS‐CoV 7b has some attenuating function [87]. By homology, SARS‐CoV‐2 7b is likely to have a similar function
